# Acute care and emergency general surgery in patients with chronic liver disease: how can we optimize perioperative care? A review of the literature

**DOI:** 10.1186/s13017-018-0194-1

**Published:** 2018-07-18

**Authors:** Michael S. Bleszynski, Alexsander K. Bressan, Emilie Joos, S. Morad Hameed, Chad G. Ball

**Affiliations:** 1 Department of Surgery, University of British Columbia, Vancouver, Canada; 2 Department of Surgery, University of Calgary, Foothills Medical Centre, 1403 - 29 Street NW, Calgary, Alberta Canada

**Keywords:** Chronic liver disease, Cirrhosis, Acute care surgery, General surgery, Portal hypertension

## Abstract

The increasing prevalence of advanced cirrhosis among operative candidates poses a major challenge for the acute care surgeon. The severity of hepatic dysfunction, degree of portal hypertension, emergency of surgery, and severity of patients’ comorbidities constitute predictors of postoperative mortality. Comprehensive history taking, physical examination, and thorough review of laboratory and imaging examinations typically elucidate clinical evidence of hepatic dysfunction, portal hypertension, and/or their complications. Utilization of specific scoring systems (Child-Pugh and MELD) adds objectivity to stratifying the severity of hepatic dysfunction. Hypovolemia and coagulopathy often represent major preoperative concerns. Resuscitation mandates judicious use of intravenous fluids and blood products. As a general rule, the most expeditious and least invasive operative procedure should be planned. Laparoscopic approaches, advanced energy devices, mechanical staplers, and topical hemostatics should be considered whenever applicable to improve safety. Precise operative technique must acknowledge common distortions in hepatic anatomy, as well as the risk of massive hemorrhage from porto-systemic collaterals. Preventive measures, as well as both clinical and laboratory vigilance, for postoperative hepatic and renal decompensation are essential.

## Background

Approximately one in every ten patients with chronic liver disease requires surgery in the final 2 years of life [1]. This commonly quoted estimation was originally reported over four decades ago, and it does not account for the expanding surgical eligibility of cirrhotic patients due to advances in both perioperative care and operative techniques for non-hepatic and hepatic operations (i.e., resection of hepatocellular carcinoma and liver transplantation). In the greater context, non-hepatic surgery is far more common and typically presents within the everyday scope of acute care surgery.

Chronic liver disease, however, imposes a significantly increased risk of postoperative morbi-mortality, largely due to sepsis and hepatic failure. In a systematic review conducted by de Goede et al., the overall risk of perioperative morbidity and mortality were 30.1 and 11.6% respectively and the coexistence of portal hypertension was found to be associated with a twofold increase in mortality [2]. In another study identifying 772 cirrhotic patients, the severity of hepatic dysfunction and emergency surgery were both major risk factors for postoperative mortality [3]. An analysis of Nationwide Inpatient Sample data describing over 22,000 patients with cirrhosis also showed an increase in adjusted mortality rates in a stepwise manner with the severity of hepatic dysfunction [4]. This was observed even for elective procedures and in the absence of portal hypertension.

Assessment and management of chronic liver disease in operative patients thus represent a relevant topic for general surgeons. More specifically, understanding the pathophysiology and clinical aspects of hepatic dysfunction and portal hypertension has a direct impact in the perioperative care of this patient population. The goal of this study is therefore to provide an updated review of relevant aspects of cirrhosis for the acute care surgeon.

A brief description of pertinent pathophysiology provides the basis of this review upon which surgical topics are expanded to include state-of-the art evidence, where available. The literature search was limited to English publications from two databases (EMBASE and PubMed) and manual search of additional references. A narrative format was utilized to better accommodate a broad comprehensive coverage of major topics of interest for the general surgeon. Our literature search was not limited to a specific question or strategy.

## Pathophysiology

Chronic hepatocyte injury is characterized by inflammation, fibrosis, and ultimately cirrhosis. Cirrhosis thus results from distortion of hepatic architecture and increased resistance to portal venous flow secondary to progressive fibrotic replacement of hepatocytes. Portal hypertension and loss of metabolic functions are the main factors that determine the clinical manifestations of cirrhosis.

The most important clinical manifestations of portal hypertension are varices and ascites. An increased pressure gradient from the post-hepatic venous system opens collateral portosystemic shunts in the esophagogastric and anorectal regions, umbilical vein, and retroperitoneum. As this process advances, visceral congestion increases, ascites accumulates, varices enlarge, and portal venous flow decelerates, or even reverses. Diminished delivery of hepatotrophic factors further impairs hepatic function via ongoing hepatocyte loss.

In surgical patient, hepatic dysfunction translates into an increased risk of infection, hemorrhage, thrombosis, and a prolonged half-life of numerous drugs (e.g., opioids and benzodiazepines). The associated imbalance of neuroendocrine mediators (e.g., vasopressin, renin-angiotensin-aldosterone system, and nitric oxide) also potentiates sodium and fluid retention, resulting in a background hyperdynamic circulation with splanchnic venous congestion and systemic vasodilation [5].

In emergency surgery, stress response to the underlying condition, anesthesia, and surgical trauma increase the risk of hepatic decompensation and associated multi-system failure. Changes in hepatic perfusion with shock and fluid shifts further compromise hepatocellular synthetic and excretory functions. In addition, endotoxemia from gram-negative sepsis potentiates platelet aggregation and creates a state of low-grade disseminated intravascular coagulation [6].

### Renal system

Activation of the renin-angiotensin-aldosterone system with hypersecretion of vasopressin acts as compensatory mechanisms to preserve arterial pressure and replenish effective circulating volume when blood is pooled in the splanchnic territory. With the progression of cirrhosis, avidity for water and sodium increases, and dilutional hyponatremia results from water retention. In advanced cases, exacerbation of these abnormalities leads to hepatorenal syndrome (HRS). HRS is characterized by rapid decline in renal function with low urinary excretion of sodium due to severe renal vasoconstriction and diminished or absent cortical perfusion. This syndrome is potentially reversible, but carries an extremely poor prognosis [7].

### Cardiovascular system

The bypass of bacterial endotoxins from the splanchnic to the systemic circulation and an increased production of nitric oxide cause peripheral vasodilation and increased cardiac output in cirrhotic patients. Along with this vasodilator-mediated hyperdynamic circulatory state, decreased myocardial contractility, accentuated in cases of chronic alcohol use, has been described. These events compromise mean arterial pressure and also impair the cardiac adaptive response to resuscitative intravenous fluid expansion.

### Respiratory system

Patients are at increased risk of hypoxia secondary to restrictive lung disease (due to ascites and pleural effusions), diffusion abnormalities due to pulmonary arterial and venous vasodilation and shunting, and pulmonary hypertension. Patients with large-volume ascites are also predisposed to bronchopulmonary aspiration.

### Central nervous system

Hepatic encephalopathy is a reversible neurological condition associated with hepatic failure and portal hypertension that may present with varying degrees of cognitive impairment, ranging from minimal changes to deep coma. This pathophysiology is driven by cerebral edema and an increased passage of ammonia through the blood-brain barrier [8]. Ammonia is produced via glutamine metabolism by enterocytes, and urea breakdown by gut flora. With impaired hepatocellular function and portosystemic shunting, ammonia is less likely to be converted to urea and therefore accumulates within the bloodstream. It eventually crosses the blood-brain barrier where it contributes to cerebral edema and other pathological processes [9].

### Coagulation

Primary hemostasis is impaired in advanced cirrhosis due to thrombocytopenia and platelet dysfunction, but it is associated with a compensatory increase in von Willebrand factor levels. Platelet abnormalities result from splenic sequestration, bone marrow suppression (secondary to alcohol, folic acid deficiency, or viral hepatitis C), decreased hepatic production of thrombopoietin, infection, and renal failure.

When activated, platelets trigger secondary hemostasis, generating thrombin to convert fibrinogen to fibrin. With the exception of von Willebrand factor, all plasma clotting factors, anticoagulants, and fibrinolytic proteins are synthesized by the liver and susceptible to advancing hepatic disease. Deficient excretion of bile salts impairs absorption of liposoluble vitamins and the synthesis of vitamin K-dependent clotting factors (factors II, VII, IX, and X). The liver also clears activated clotting factors, anticoagulants, and fibrinolytic proteins. Such widespread effects on coagulation and anticoagulation translate into an unpredictably increased risk of both bleeding and thrombosis.

## Preoperative assessment and management

Hypovolemia, coagulopathy, and thrombocytopenia are the major concerns during preoperative assessment and resuscitation. Goal-directed fluid administration and use of blood products (Table 1) should be guided, where possible, by hemodynamic monitoring [10]. Low urine output should be interpreted with caution. Oliguria from hormonal and inflammatory changes associated with cirrhosis and the underlying emergency might mislead the clinician to volume overload the patient. Excessive crystalloid and blood product infusion, in turn, can precipitate respiratory failure and variceal hemorrhage.

**Table 1:** Management of perioperative coagulopathy in chronic liver disease

Treatment	Comment	Dosing principles
Vitamin K	IV vitamin K can be given for patients who are severely malnourished or have malabsorption, secondary to biliary obstruction, bile salt deficiency, or use of broad spectrum antibiotics.	The recommended dose in 10 mg IV daily for 3 days prior to surgery.
Fresh frozen plasma (FFP)	The correction of INR with FFP has not been shown to decrease the risk of bleeding in cirrhotics. It is not recommended to empirically transfuse FFP for elevated INR or prothrombin time. Excessive use of FFP can lead to significant complications such as volume expansion, infection, and transfusion-associated lung injury (TRALI).	If the patient is clinically bleeding, it is recommended to transfuse FFP, at a dose of 10–15 ml/kg.
Platelets	Platelet transfusion should be considered in active bleeding if platelet levels are below 50,000.	The recommended dose is 1 unit per 10 kg body weight.
Cryoprecipitate	Hypofibrinogenemia (≤ 100 mg/dl) should be corrected with cryoprecipitate.	The recommended dose is one bag (10 units) per 10 kg of body weight.
Tranexemic acid	Tranexemic acid, an anti-fibrinolytic agent, should be used in patients with hyperfibrinolysis diagnosed by thromboelastography or patients with intractable bleeding.	A loading dose of 10 mg/kg is given, repeated three times for 2–8 days.
DDAVP	DDAVP is an analogue of vasopressin. It releases vWF and factor VIII. Despite the high levels of vWF in cirrhosis, DDAVP has been shown to decrease bleeding time in those patients.	The recommended dose is 300 μg IV.
rfVIIa	rfVIIa has a high cost, transient effect, and thrombotic complications. It has been shown to reduce bleeding in the placement of intracranial pressure monitoring devices but not in any other surgical procedure. Its clinical indications are limited.	If used, the dose is 40 μg/kg.

Conventional coagulation tests, which commonly focus on measuring defects in specific hemostatic pathways, often do not provide an accurate picture of the overall clotting ability within cirrhosis. Furthermore, these tests are poor predictors of the risk of hemorrhage versus thrombosis [11]. Given that thromboelastography evaluates the viscoelastic properties of blood and therefore provides a comprehensive evaluation of the clotting process, its use has been recommended in the global assessment of clot formation, strength, and dissolution. In a prospective study, thromboelastography differentiated cirrhotic patients from healthy volunteers (AUC = 0.921, *p* < 0.001), while conventional coagulation tests did not [12]. A growing body of literature supports the use of thromboelastography in liver transplantation [13, 14], but more studies are still necessary for many other diagnostic tests [15]. Other coagulation studies such as sonorheometry, international normalized ratio calibrated for cirrhosis, and coagulation-like thrombin generation time have also been recommended, but still lack definitive prospective validation for cirrhotic patients [11].

Electrolyte imbalances (hypokalemia, hypocalcemia, hypomagnesemia, and dilutional hyponatremia) should be monitored and corrected. A high index of suspicion for malnutrition and micronutrient deficiencies (folate, vitamins A, D, E, K, and complex B) needs to be maintained. Early supplementation of these nutrients is also recommended, particularly in alcoholic liver disease. Hypoglycemia, from impaired gluconeogenesis, should also be expected and promptly corrected. Blood lactate level, a specific marker of tissue ischemia, can be significantly elevated in the context of chronic liver disease due to impaired hepatic clearance. Nevertheless, the trend in serial measurements in serum lactate in response to resuscitation may be a useful adjunct in hemodynamic assessment. Mild increases in aminotransferases (AST and ALT) are commonly noted in chronic liver disease, reflecting active hepatic injury. Marked elevation (greater than 1000 IU/l), however, suggests acute viral, alcoholic, or ischemic hepatitis. In asymptomatic patients with three or more fold increases in ALT/AST levels or any elevation in bilirubin, the incidence of undiagnosed cirrhosis ranges from 6 to 34%.

The precise etiology of the underlying hepatic disease mandates specific treatment measures. Thiamine replacement with glucose infusion is indicated to prevent progression of Wernicke encephalopathy. The risk of symptoms from alcohol withdrawal also mandates supportive measures, as well as multivitamins. Patients on chronic steroids for autoimmune hepatitis should receive a stress-dose adjustment in the perioperative period. Finally, delays in restarting anti-viral therapy for hepatitis should be avoided.

### Scoring systems for cirrhosis: Child and MELD systems

The Child-Turcotte classification system was initially proposed in 1964 to predict mortality after portosystemic shunt surgery [16]. It was then modified in 1972 by Pugh et al., when the nutritional status criterion was replaced by prothrombin time (or INR) [17]. The Child-Pugh classification relies on three objective laboratory (albumin level, bilirubin level, and prothrombin time) and two subjective clinical (severity of ascites and encephalopathy) criteria to stratify patients into three classes (A, B, or C; with C representing the most advanced cases). It is currently utilized in a variety of surgical and medical scenarios. Predicted mortality varies from 10% for Child-Pugh A, 30% for Child-Pugh B, and up to 80% for Child-Pugh C [18, 19]. Its discriminatory ability, however, is extremely compromised by inter-observer variability of the subjective criteria, and the stratification of a wide range of laboratory values into only three risk groups.

The sentinel publication of the Model of End-Stage Liver Disease (MELD) in 2000 introduced a more accurate scoring system to predict mortality after insertion of transjugular intra-hepatic portosystemic shunts (TIPS), based on bilirubin, creatinine, and international normalized ratio (INR) values [20]. In the original publication, a MELD score < 8 was predictive of good post-TIPS survival, whereas a MELD score > 18 translated into significantly greater mortality. Since then, a number of series have validated the MELD system for different operations, with slightly variable reported cutoff values for poor outcomes. In a review of the literature, Hanje and colleagues concluded that elective abdominal surgery could be recommended for MELD scores < 10, but should be strongly discouraged for a MELD scores > 15 [21]. The current major utility of the MELD system, however, has been demonstrated in prioritizing patients on liver transplantation waiting lists [22]. Recently, the superiority of a revised MELD system incorporating sodium levels has been suggested [23], but is awaiting further validation [24].

In non-transplant patients, MELD risk stratification is also often preferred to Child-Pugh scoring due to its more objective and detailed risk stratification with a continuum of possible scores. Unfortunately, and similar to the limitations associated with the Child-Pugh score, the MELD is not specific to patients with surgical emergencies.

### Imaging exams

Morphologic findings of early cirrhosis include hepatomegaly, as well as widening of the *porta hepatis*, umbilical fissure, and pericholecystic space. As cirrhosis advances, hepatomegaly evolves into the typical nodular shrunken liver with atrophy of segment 4 and the right lobe, as well as hypertrophy of segments 2 and 3 and the caudate lobe. Bright and coarse nodular texture with surface nodularity (most commonly noticed on the undersurface of the liver) can be demonstrated on bedside ultrasonography [25]. Dysplastic and regenerative nodules are difficult to appreciate on CT due to their small size and isointensity. However, the identification of nodules, particularly if they are hyper-enhancing, on the arterial phase, should raise suspicion for an incidental hepatocellular carcinoma [26].

Vascular manifestations of cirrhosis include signs of hepatic perfusion abnormalities and portal hypertension. Progressive hepatic fibrosis increases resistance to both arterial and venous inflow. While the portal venous system may decompress through portosystemic collaterals, there is no equivalent alternative for hepatic arterial inflow. Intra-hepatic vascular communications thus develop in the hepatic sinusoids, *vasa vasorum* of portal vein, and peribiliary capillaries to shunt arterial blood into the portal venous system [27]. These changes present on cross-sectional imaging as heterogeneous delayed enhancement of hepatic parenchyma, sometimes mixed with geographic areas of arterialization. Evidence of overt portal hypertension can also be found on imaging (esophageal, gastric, and umbilical varices, prominent left gastric vein and tributaries, splenomegaly, and ascites).

## Intraoperative considerations

Surgical access (open or laparoscopic) and placement of retractors should be planned to avoid engorged abdominal wall veins and to optimize surgical exposure in anticipation to the most critical operative steps. Despite the lack of definitive evidence [28], the authors believe that bipolar and ultrasonic energy devices, mechanical vascular staplers, and topical hemostatics are useful adjuncts in attempting to decrease both operating time and blood loss. This seems particularly true for the surgeon coming through the abdominal wall with associated dilated veins. Use of a concurrent ligating and cutting energy instrument in a careful and cautious manner can help prevent undue blood loss. Postoperative coagulopathy can easily facilitate bleeding from initially minor sources, so extra attention to hemostasis is required.

Utilization of intra-abdominal drains to help control postoperative ascites and prevent surgical wound complications is a controversial topic. While a more restrict policy for placement of surgical abdominal drains has gained growing support in the literature [29], its safety in the context of cirrhosis yet remains poorly explored. Better control of postoperative ascites and potential associated surgical wound complications presents a compelling rationale for prophylactic drainage, but the risk of contamination of ascites and increased postoperative fluid shifts should be taken into account. In fact, increased morbidity due to wound complications was demonstrated in cirrhotic patients randomized to prophylactic drainage after liver resection in a small clinical trial conducted in the early 2000s [30]. It is the opinion of the authors of this review that routine use of surgical drains should be avoided in cirrhotic patients, and if indicated, early removal should be aggressively pursued.

As a general rule, the most expeditious and least invasive operation should be utilized, including a laparoscopic approach where feasible and safe. The safety of laparoscopy in cirrhotic patients has been historically challenged due to the theoretical risks of hemorrhage from abdominal wall varices during port placement, detrimental effects of pneumoperitoneum on hepatic perfusion, and technical limitations to approaching intraoperative hemorrhage. These concerns, however, have been vastly mitigated over the years, with multiple reports now attesting to the safety of laparoscopy, as well as suggesting some useful technical tips (Table 2).

**Table 2:** Laparoscopic strategies in advanced liver disease

Port placement	As a general rule, an open Hasson technique is recommended for entry into the peritoneal cavity [31]. To avoid abdominal wall varices from the umbilical vein and falciform ligament, several authors have recommended port placement to the right of the midline, especially the subxiphoid port in laparoscopic cholecystectomy [32, 33].
Pneumoperitoneum	Blunting of the hepatic arterial buffer response is a theoretical risk. There is no report in the literature of liver failure related to pneumoperitoneum in a cirrhotic patient [34, 35]. Some authors reduce their intra-abdominal pressure in cirrhotic patients [36].
Bleeding risk	There are few reports of bleeding during laparoscopic procedures in cirrhotic patients. Cobb et al. describes an 8% transfusion rate, with only one patient requiring transfusion for bleeding [37]. In a retrospective study of 68 patients undergoing laparoscopic cholecystectomy, only one patient received a blood transfusion [35]. Laparoscopic hemostatic devices such as ultrasound knife, ligasure, and harmonic scalpel can significantly improve hemostasis and are recommended by many authors [32, 36].
Conversion	The published conversion rate for laparoscopic cholecystectomy is between 4 and 6%, which is similar to the non-cirrhotic patient population [32, 35–37].
Immune function	Li et al. report a reduced risk of bacterial seeding with laparoscopy, with subsequent decreased risk in spontaneous bacterial peritonitis [36].

The superiority of laparoscopic over open cholecystectomy in cirrhotic patients has been demonstrated in terms of operative blood loss, surgical time, postoperative pain, morbidity, and hospital length of stay [31, 32]. Technical difficulties must be expected in retracting the liver and identifying anatomic landmarks due to hepatic distortion.

In cirrhotic patients, an increased risk of intraoperative hemorrhage should be expected. In laparoscopic surgery, utilization of additional ports and meticulous operative technique assist in preventing iatrogenic injuries. Venous hemorrhage can be temporized by brief increases in pneumoperitoneum pressure and compression with sponges. If application of electrocautery is contemplated for gallbladder bed bleeding, a high setting (100 units on spray) and precise contact to the site of bleeding is recommended. In cases where this technique does not arrest ongoing bleeding, placement of a clip immediately beside the site of hemorrhage (i.e., into the liver in a perpendicular manner) can be helpful as an ignition tool for cauterization.

Laparoscopy has also been recommended for cirrhotic patients with appendicitis. Tsugawa and colleagues compared open (25 patients) to laparoscopic appendectomy (15 patients) in a retrospective series. Laparoscopy compared favorably with open surgery with respect to blood loss, ascites formation, wound complications, hospital length of stay, postoperative pain, and liver function [33].

Ascites predisposes patients with chronic liver disease to ventral hernias. Up to 20% of all patients with cirrhotic ascites develop umbilical hernias [34]. Surgical indications are debatable in non-complicated cases due to the high risk of postoperative morbidity and recurrence (60%) in patients with persistent ascites [35]. Conservative management, however, imposes a risk of hernia strangulation and rupture of overlying skin, with even poorer surgical outcomes. Once an emergency occurs, prompt surgical treatment and clinical management of ascites are mandatory. Concerns about the increased risk of surgical site infection, ascites leakage, and mesh displacement mandate judicious planning of the repair technique by the acute care surgeon. To minimize the risk of these complications, when a repair with mesh is indicated, pre-peritoneal placement has been favored over an onlay technique. A laparoscopic approach has also been proven safe in multiple surgical series [35–37].

## Postoperative issues

Much of the resuscitative and therapeutic effort initiated preoperatively, including volume resuscitation, correction of electrolyte abnormalities, and coagulopathy, must be continued in the postoperative period. The surgical team should also maintain a high degree of vigilance for evidence of hepatic decompensation. Hepatic failure might simply result from the inflammatory response to surgery and underlying emergency condition, but the possibility of precipitating factors (e.g., constipation, fluid and electrolyte abnormalities, gastrointestinal hemorrhage, and spontaneous bacterial peritonitis) should always be considered.

### Ascites

Large-volume ascites after abdominal surgery increases the risk of abdominal wall dehiscence and herniation. It also predisposes the patient to atelectasis, aspiration, and pneumonia. Associated fluid shifts might precipitate electrolyte imbalances, hypovolemia, and acute kidney injury. Management is based on sodium restriction and judicious use of diuretics, with close monitoring of electrolytes and renal function. Therapeutic paracentesis should be reserved for refractory cases and limited to symptomatic relief. A high index of suspicion for spontaneous bacterial peritonitis is also required.

### Hepatic encephalopathy

Other possible etiologies of global neurologic decline should be investigated (e.g., hypoxia, hypercapnia, hypoglycemia, uremia, medications, *delirium tremens*, hypoactive delirium, seizures, intracranial hemorrhage). Elevated blood ammonia level (found in over 90% of cases) corroborates the clinical diagnosis, but correlation with the actual disease severity is poor. It should also be noted that monitoring blood ammonia levels is inferior to clinical assessment, and it is not recommended in asymptomatic patients [37]. Benzodiazepines should be avoided and replaced with haloperidol when chemical restraint is indicated.

The classic protein-restricted diet does not find support in the current literature [38] because most patients do not tolerate high-food/calorie intake. Furthermore, malnutrition is a much more significant concern. Despite ongoing debate, branched amino acid supplementation may also be beneficial [39]. Other adjuncts, such as cathartics and oral antibiotics, are recommended in an attempt to decrease the intestinal production of ammonia due to bacterial overgrowth.

Lactulose, a non-absorbable sugar that causes osmotic diarrhea, has been traditionally used to treat hepatic encephalopathy. It acidifies the colon and promotes the conversion of ammonia to ammonium, which is not reabsorbed [40]. Lactulose dosing should be titrated to two to three bowel movements per day. Known side effects of this therapy include electrolyte imbalances, nausea, and bloating.

Rifaximin, a semisynthetic drug derivative of rifampin, was originally employed as a second-line treatment for hepatic encephalopathy. In 2012, a meta-analysis of 12 randomized controlled trials stated that rifaximin had similar effectiveness compared to lactulose, with fewer side effects [41]. A more recent, larger meta-analysis has also further supported its use [42], although the dominant limitation remains its high cost when compared to lactulose.

### Hepatorenal syndrome

Euvolemia and electrolyte homeostasis should be maintained under strict clinical and laboratory monitoring. Acute kidney injury in the absence of hemodynamic instability, use of nephrotoxic drugs, or parenchymal renal disease suggests the diagnosis of hepatorenal syndrome. Additional diagnostic criteria include no improvement in renal function after volume expansion with albumin and diuretic withdrawal. Treatment of hepatorenal syndrome requires the use of splanchnic vasoconstrictors (terlipressin, noradrenalin, or midodrine) and albumin infusion. Improved renal function has been demonstrated with medical treatment [10], but a mortality benefit is only ultimately achieved with liver transplantation. TIPS placement and renal and hepatic replacement therapies can be indicated as bridging strategies.

### Variceal hemorrhage

Perioperative volume overload can increase portal pressure and precipitate bleeding from esophageal varices. Other possible contributing factors include postoperative hepatic dysfunction and portal venous thrombosis. Variceal hemorrhage represents a medical emergency to be managed in the intensive care unit [43].

Resuscitative measures to preserve the respiratory and circulatory systems should be promptly initiated. A restrictive transfusion strategy aiming at hemoglobin levels of 7–9 g/dl has been associated with decreased re-bleeding and mortality rates [44]. Antibiotic prophylaxis with ceftriaxone for up to 7 days is indicated to address the increased risk of bacterial infections and also to decrease the risk of re-bleeding and mortality [45]. Infusion of vasoactive agents (somatostatin, terlipressin, and octreotide) for 2 to 5 days has been associated with lower mortality and transfusion requirements [46].

An urgent endoscopy should be completed no longer than 12 h from presentation [43]. Elastic band ligation represents the first-line endoscopic intervention for esophageal varices, but sclerotherapy is an excellent alternative for difficult cases [47]. Elastic band ligation has proven more effective than sclerotherapy in terms of the number of required sessions, as well as re-bleeding and mortality rates [48, 49]. A non-specific beta-blocker (propranolol or nadolol) is indicated after the vasoactive infusion is discontinued.

Balloon tamponade is reserved for temporary control of ongoing variceal bleeding while definitive therapy is being arranged. TIPS should be considered pre-emptively in high-risk patients, or in refractory or recurrent hemorrhage, as improved mortality rates have been observed [50].

Table 3 summarizes current recommendations for the management of hemorrhage from esophageal varices.

**Table 3:** Management of variceal hemorrhage

Blood product transfusion: aim at hemoglobin levels between 7 and 9 g/dl.	
Antibiotic prophylaxis: ceftriaxone 1 g/dl, no longer than 7 days.	
Vasoactive agents (octreotide, terlipressin, somatostatin): bolus followed by infusion for 2 to 5 days.	
Endoscopy: no longer than 12 h from presentation.	
Start non-specific beta-blocker (propranolol, nadolol): once vasoactive agent is discontinued.	
Consider TIPS: high-risk patients, refractory or recurrent hemorrhage.	

### Venous thromboembolism prophylaxis

Coagulation disorders of cirrhosis confer an increased risk of both venous thromboembolism and hemorrhage. As previously indicated, conventional coagulation tests do not reflect these risks, and an increased INR is not necessarily protective for thromboembolic events. Although detailed guidelines remain unavailable, thromboprophylaxis is recommended in most patients, and certainly in high-risk situations.

## Conclusions

Chronic liver disease in the surgical patient directly affects perioperative care. In acute care surgery, cirrhosis and the postoperative inflammatory response confound the boundaries between low effective circulating volume with renal failure and anasarca with respiratory failure. Perioperative care should contemplate specific topics such as (1) severity stratification for hepatic dysfunction, (2) imaging findings of portal hypertension, (3) management of coagulation disorders, and (4) vigilance for postoperative hepatic failure (e.g., ascites, jaundice, hepatic encephalopathy, hepatorenal syndrome, and variceal hemorrhage) and possible precipitating factors.

## Abbreviations

ALT: Alanine aminotransferase
AST: Aspartate aminotransferase
CT: Computed tomography
HRS: Hepatorenal syndrome
INR: International normalized ratio
MELD: Model of End-Stage Liver Disease
TIPS: Transjugular intra-hepatic portosystemic Shunt

## References

[1] Jackson FC, Christophersen EB, Peternel WW, Kirimli B (1968). Preoperative management of patients with liver disease. Surg Clin North Am.

[2] de Goede MSc B, MD PJK, Professor JFLM, Professor HJMM, Professor GKM (2012). Morbidity and mortality related to non-hepatic surgery in patients with liver cirrhosis: a systematic review. Best Pract Res Clin Gastroenterol.

[3] Teh SH, Nagorney DM, Stevens SR, Offord KP, Therneau TM, Plevak DJ, Talwalkar JA, Kim WR, Kamath PS (2007). Risk factors for mortality after surgery in patients with cirrhosis. Gastroenterology.

[4] Csikesz NG, Nguyen LN, Tseng JF, Shah SA (2009). Nationwide volume and mortality after elective surgery in cirrhotic patients. J Am Coll Surg.

[5] Moore CM (2013). Cirrhotic ascites review: pathophysiology, diagnosis and management. World J Health.

[6] Blasi A (2015). Coagulopathy in liver disease: lack of an assessment tool. World J Gastroenterol.

[7] Arroyo V, Terra C, Gines P (2007). Advances in the pathogenesis and treatment of type-1 and type-2 hepatorenal syndrome. J Hepatol.

[8] Patidar KR, Bajaj JS (2015). Covert and overt hepatic encephalopathy: diagnosis and management. Clin Gastroenterol Hepatol.

[9] Dhiman RK (2013). Gut microbiota and hepatic encephalopathy. Metab Brain Dis.

[10] Davenport A, Ahmad J, Al-Khafaji A, Kellum JA, Genyk YS, Nadim MK (2012). Medical management of hepatorenal syndrome. Nephrol Dial Transplant.

[11] Amarapurkar PD, Amarapurkar DN (2011). Management of coagulopathy in patients with decompensated liver cirrhosis. Int J Hepatol.

[12] Branco BC, Inaba K, Ives C, Okoye O, Shulman I, David JS, Schöchl H, Rhee P, Demetriades D (2014). Thromboelastogram evaluation of the impact of hypercoagulability in trauma patients. Shock.

[13] Coakley M, Reddy K, Mackie I, Mallett S (2006). Transfusion triggers in orthotopic liver transplantation: a comparison of the thromboelastometry analyzer, the thromboelastogram, and conventional coagulation tests. J Cardiothorac Vasc Anesth.

[14] Feltracco P, Barbieri S, Cillo U, Zanus G, Senzolo M, Ori C (2015). Perioperative thrombotic complications in liver transplantation. World J Gastroenterol.

[15] De Pietri L, Bianchini M, Montalti R, De Maria N, Di Maira T, Begliomini B, Gerunda GE, di Benedetto F, Garcia-Tsao G, Villa E. Thrombelastography-guided blood product use before invasive procedures in cirrhosis with severe coagulopathy: A randomized, controlled trial. Hepatology. 2016;63(2):566–73. 10.1002/hep.28148 26340411

[16] Child CG, Turcotte JG, Child CG (1964). Surgery and portal hypertension. The liver and portal hypertension.

[17] Pugh RN, Murray-Lyon IM, Dawson JL, Pietroni MC, Williams R (1973). Transection of the oesophagus for bleeding oesophageal varices. Br J Surg.

[18] Garrison RN, Cryer HM, Howard DA, Polk HC (1984). Clarification of risk factors for abdominal operations in patients with hepatic cirrhosis. Ann Surg.

[19] Mansour A, Watson W, Shayani V, Pickleman J (1997). Abdominal operations in patients with cirrhosis: still a major surgical challenge. Surgery.

[20] Malinchoc M, Kamath PS, Gordon FD, Peine CJ, Rank J, ter Borg PC (2000). A model to predict poor survival in patients undergoing transjugular intrahepatic portosystemic shunts. Hepatology.

[21] Hanje AJ, Patel T (2007). Preoperative evaluation of patients with liver disease. Nat Clin Pract Gastroenterol Hepatol.

[22] Wiesner RH, McDiarmid SV, Kamath PS, Edwards EB, Malinchoc M, Kremers WK, Krom RA, Kim WR (2001). MELD and PELD: application of survival models to liver allocation. Liver Transpl.

[23] Biggins SW, Kim WR, Terrault NA, Saab S, Balan V, Schiano T, Benson J, Therneau T, Kremers W, Wiesner R (2006). Evidence-based incorporation of serum sodium concentration into MELD. Gastroenterology.

[24] Causey MW, Steele SR, Farris Z, Lyle DS, Beitler AL (2012). An assessment of different scoring systems in cirrhotic patients undergoing nontransplant surgery. Am J Surg.

[25] Yeom SK, Lee CH, Cha SH, Park CM (2015). Prediction of liver cirrhosis, using diagnostic imaging tools. World J Hepatol.

[26] Sangster GP, Previgliano CH, Nader M, Chwoschtschinsky E, Heldmann MG (2013). MDCT imaging findings of liver cirrhosis: spectrum of hepatic and extrahepatic abdominal complications. HPB Surg.

[27] Wachsberg RH, Bahramipour P, Sofocleous CT, Barone A (2002). Hepatofugal flow in the portal venous system: pathophysiology, imaging findings, and diagnostic pitfalls. Radiographics.

[28] Moggia E, Rouse B, Simillis C, Li T, Vaughan J, Davidson BR (2016). Methods to decrease blood loss during liver resection: a network meta-analysis. Cochrane Database Syst Rev.

[29] Wong-Lun-Hing EM, van Woerden V, Lodewick TM, Bemelmans MHA, Olde Damink SWM, Dejong CHC (2017). Abandoning prophylactic abdominal drainage after hepatic surgery: 10 years of no-drain policy in an enhanced recovery after surgery environment. Dig Surg.

[30] Liu CL, Fan ST, Lo CM, Wong Y, Ng IO, Lam CM (2004). Abdominal drainage after hepatic resection is contraindicated in patients with chronic liver diseases. Ann Surg.

[31] El-Awadi S, El-Nakeeb A, Youssef T, Fikry A, El-Hamed TMA, Ghazy H, Foda E, Farid M (2009). Laparoscopic versus open cholecystectomy in cirrhotic patients: a prospective randomized study. Int J Surg.

[32] Puggioni A, Wong LL (2003). A metaanalysis of laparoscopic cholecystectomy in patients with cirrhosis. J Am Coll Surg.

[33] Tsugawa K, Koyanagi N, Hashizume M, Tomikawa M, Ayukawa K, Akahoshi K, Sugimachi K (2001). A comparison of an open and laparoscopic appendectomy for patients with liver cirrhosis. Surg Laparosc Endosc Percutan Tech.

[34] Belghiti J, Durand F (1997). Abdominal wall hernias in the setting of cirrhosis. Semin Liver Dis.

[35] Belli G, D'Agostino A, Fantini C, Cioffi L, Belli A, Russolillo N, Langella S (2006). Laparoscopic incisional and umbilical hernia repair in cirrhotic patients. Surg Laparosc Endosc Percutan Tech.

[36] Yu BC, Chung M, Lee G (2015). The repair of umbilical hernia in cirrhotic patients: 18 consecutive case series in a single institute. Ann Surg Treat Res.

[37] James J, Liou IW (2015). Comprehensive care of patients with chronic liver disease. Med Clin North Am.

[38] Cabral CM, Burns DL (2011). Low-protein diets for hepatic encephalopathy debunked: let them eat steak. Nutr Clin Pract.

[39] Gluud LL, Dam G, Les I, Cordoba J, Marchesini G, Borre M, Aagaard NK, Vilstrup H (2015). Branched-chain amino acids for people with hepatic encephalopathy. Cochrane Database Syst Rev.

[40] Patil DH, Westaby D, Mahida YR, Palmer KR, Rees R, Clark ML, Dawson AM, Silk DB (1987). Comparative modes of action of lactitol and lactulose in the treatment of hepatic encephalopathy. Gut.

[41] Eltawil KM (2012). Rifaximin vs conventional oral therapy for hepatic encephalopathy: a meta-analysis. World J Gastroenterol.

[42] Kimer N, Krag A, Møller S, Bendtsen F, Gluud LL (2014). Systematic review with meta-analysis: the effects of rifaximin in hepatic encephalopathy. Aliment Pharmacol Ther.

[43] Garcia-Tsao G, Abraldes JG, Berzigotti A, Bosch J (2017). Portal hypertensive bleeding in cirrhosis: risk stratification, diagnosis, and management: 2016 practice guidance by the American Association for the study of liver diseases. Hepatology.

[44] Villanueva C, Colomo A, Bosch A, Concepcion M, Hernandez-Gea V, Aracil C (2013). Transfusion strategies for acute upper gastrointestinal bleeding. N Engl J Med.

[45] Chavez-Tapia NC, Barrientos-Gutierrez T, Tellez-Avila F, Soares-Weiser K, Mendez-Sanchez N, Gluud C (2011). Meta-analysis: antibiotic prophylaxis for cirrhotic patients with upper gastrointestinal bleeding—an updated Cochrane review. Aliment Pharmacol Ther.

[46] Wells M, Chande N, Adams P, Beaton M, Levstik M, Boyce E (2012). Meta-analysis: vasoactive medications for the management of acute variceal bleeds. Aliment Pharmacol Ther.

[47] Bari K, Garcia-Tsao G (2012). Treatment of portal hypertension. World J Gastroenterol.

[48] Avgerinos A, Armonis A, Stefanidis G, Mathou N, Vlachogiannakos J, Kougioumtzian A, Triantos C, Papaxoinis C, Manolakopoulos S, Panani A (2004). Sustained rise of portal pressure after sclerotherapy, but not band ligation, in acute variceal bleeding in cirrhosis. Hepatology.

[49] Villanueva C, Piqueras M, Aracil C, Gomez C, Lopez-Balaguer JM, Gonzalez B, Ballego A, Torras X, Soriano G, Sáinz S (2006). A randomized controlled trial comparing ligation and sclerotherapy as emergency endoscopic treatment added to somatostatin in acute variceal bleeding. J Hepatol.

[50] Garcia-Pagan JC, Di Pascoli M, Caca K, Laleman W, Bureau C, Appenrodt B, Luca A, Zipprich A, Abraldes JG, Nevens F (2013). Use of early-TIPS for high-risk variceal bleeding: results of a post-RCT surveillance study. J Hepatol.

